# Defining the hospital night: deriving and validating electronic health record-based measures of the inpatient sleep experience

**DOI:** 10.1093/sleep/zsag164

**Published:** 2026-06-15

**Authors:** Sullafa Kadura, Samia H Lopa, Mark Oldham, Lisa Pink, Diego R Mazzotti, Sanjay R Patel

**Affiliations:** Department of Medicine, University of Rochester Medical Center, Rochester, NY, United States; University of Rochester Health Lab, University of Rochester Medical Center, Rochester, NY, United States; Center for Sleep and Cardiovascular Outcomes Research, Division of Pulmonary, Allergy, Critical Care, and Sleep Medicine, University of Pittsburgh, Pittsburgh, PA, United States; Department of Biostatistics and Computational Biology, University of Rochester, Rochester, NY, United States; Department of Psychiatry, University of Rochester Medical Center, Rochester, NY, United States; University of Rochester Health Lab, University of Rochester Medical Center, Rochester, NY, United States; Division of Medical Informatics, Department of Internal Medicine, University of Kansas Medical Center, Kansas City, KS, United States; Division of Pulmonary Critical Care and Sleep Medicine, Department of Internal Medicine, University of Kansas Medical Center, Kansas City, KS, United States; Center for Sleep and Cardiovascular Outcomes Research, Division of Pulmonary, Allergy, Critical Care, and Sleep Medicine, University of Pittsburgh, Pittsburgh, PA, United States

**Keywords:** inpatient sleep, electronic health record, sleep opportunity, sleep disruption, patient–staff interaction, hospital, TUSO

## Abstract

**Study Objectives:**

To identify electronic health record (EHR)–derived sleep measures associated with the inpatient sleep experience during hospitalization.

**Methods:**

We recruited 247 inpatients who completed the Karolinska Sleep Diary and the Potential Hospital Sleep Disruptions and Noise Questionnaire. Using EHR data, we derived three measures: longest uninterrupted sleep opportunity (LUSO), total uninterrupted sleep opportunity (TUSO), and patient–staff interactions (PSIs) calculated within two timeframes: a fixed window (23:00–06:00) and a patient-defined window (lights out to final awakening). We examined the associations between EHR-derived measures calculated within each window and sleep experience measures.

**Results:**

Sleep duration correlated more strongly with patient-defined TUSO (*r* = 0.63, *p* < .001) than with fixed TUSO (*r* = 0.27, *p* < .001). Ratings of sleep continuity, refreshing sleep, and sound sleep were also associated with patient-defined TUSO (TUSO *r* = 0.15–0.23; all *p* < .05), supporting its construct validity as an indicator of sleep quality. Sleep disruption ratings were associated with EHR-derived measures of overnight activity, including PSI counts (*r* = 0.35 within fixed windows vs. *r* = 0.29 within patient-defined windows; both *p* < .001).

**Conclusions:**

EHR-derived measures of sleep opportunity and interruption provide scalable tools for characterizing the inpatient sleep experience using routinely collected clinical data. Measures calculated within patient-defined windows show stronger associations with sleep duration and quality, while PSIs calculated within both window types show similar associations with perceived sleep disruption from overnight clinical workflows. These complementary approaches may inform strategies to reduce unnecessary nighttime disturbances and improve sleep in hospitalized patients.

**Statement of Significance**

Poor sleep is a near-universal complaint among hospitalized patients, yet methods for measuring inpatient sleep remain limited. Our study demonstrates that measures of sleep opportunity and disruption can be derived from routinely documented data in the electronic health record (EHR). Measures calculated within patient-defined sleep windows show stronger alignment with sleep duration and quality, whereas measures derived from both patient-defined and fixed workflow-defined windows capture sleep disruption associated with patient–staff interactions. Knowledge gaps remain in determining how these measures relate to patient recovery, safety, and satisfaction. Future work should investigate how EHR-based sleep measures can be leveraged to facilitate system-level interventions aimed at improving sleep in hospital settings.

## Graphical Abstract

**Graphical Abstract:**
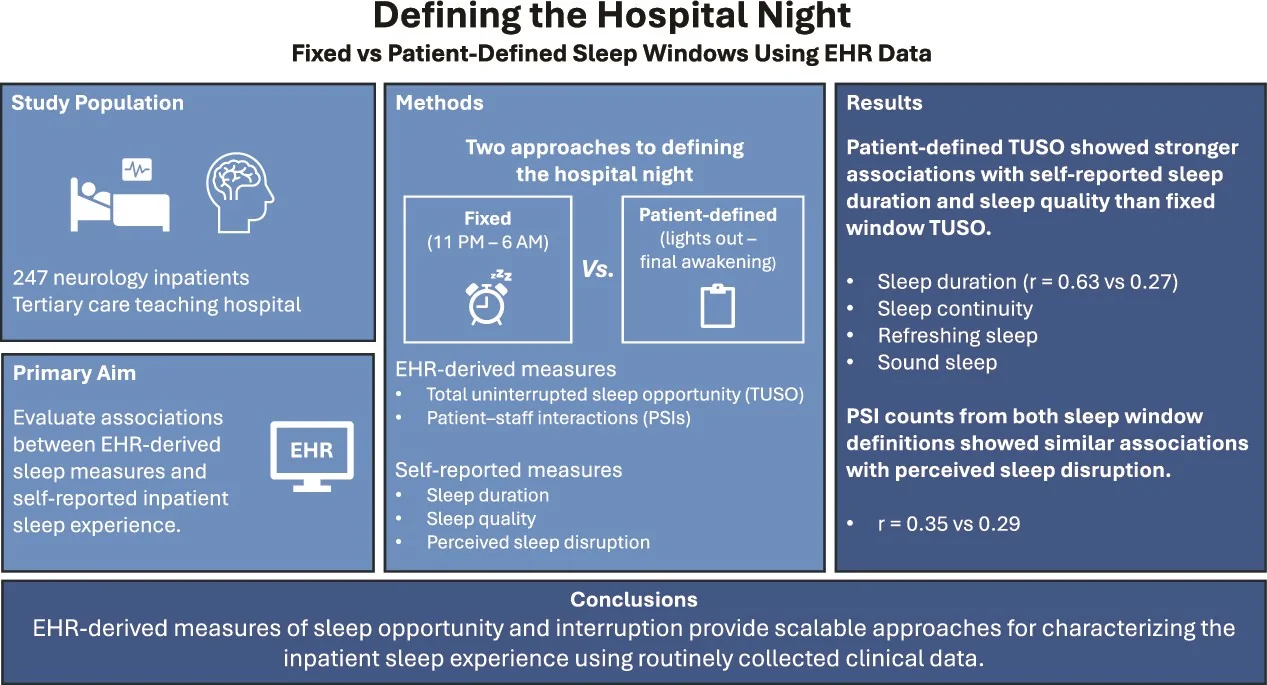

## Introduction

Sleep in the hospital is often short, disrupted, and of poor quality, contributing to delayed recovery, increased risk of complications, and lower patient satisfaction [1–3]. Staff frequently interact with patients at night, which patients describe as disruptive to their sleep [4, 5]. Despite the clinical consequences of poor sleep, hospital workflows often prioritize operational efficiency over preserving patients’ sleep, leading to fragmented, insufficient sleep [6].

Measuring inpatient sleep is challenging. While polysomnography is the gold standard, it is impractical for routine hospital use due to its technical complexity, resource-intensive demands, and poor acceptability among inpatients already burdened by acute illness. Actigraphy, though less burdensome, tends to overestimate sleep quantity and efficiency in hospitalized patients who spend sustained periods in bed [7]. As a result, inpatient sleep disturbances are often inferred through indirect observations or left unmeasured.

Electronic health record (EHR)–based methods have recently emerged as an alternative for estimating patients’ sleep experience, repurposing routine documentation of patient–staff interactions (PSIs) (e.g. vital signs, neurological checks, medication administrations, and testing) to estimate sleep opportunities and interruptions [8]. While these approaches offer a scalable way to quantify inpatient sleep, it is unclear whether these measures correlate with patients’ actual sleep experiences.

Prior work in a cardiac surgery cohort established associations between EHR-derived sleep metrics and sleep quality, as measured by the Richards–Campbell Sleep Questionnaire [9]. However, that study and all other published studies to date have defined sleep opportunity using fixed nighttime windows (e.g. 11 pm to 6 am), often without providing a rationale for the window chosen [8–12]. Therefore, it remains uncertain whether these fixed windows accurately reflect patients’ actual sleep timing or hospital workflow patterns.

Our study addresses these gaps by evaluating the association between EHR-derived sleep measures and patients’ sleep experiences in an inpatient neurology population. Further, we evaluate the performance of two EHR-based sleep window approaches: measures calculated within fixed, workflow-defined windows, and measures calculated within patient-defined sleep windows. By incorporating inpatients’ specified sleep timing, we evaluate whether fixed windows adequately capture sleep opportunities for calculating EHR-derived sleep measures and their relationship to the inpatient sleep experience.

## Materials and methods

### Study population and study design

We analyzed data from a cohort of 247 neurology inpatients who participated in a research study examining the relationship between nighttime disruptions and sleep quality from April 2021 to October 2022. Study design and survey administration procedures have been detailed previously [5]. In brief, eligible participants were adults (≥18 years) admitted to the inpatient neurology unit of a tertiary care hospital who had spent at least one prior night on the unit before survey administration, in order to reduce the potential influence of the first-night effect associated with sleeping in an unfamiliar environment. Participants were excluded if they were transferred from the intensive care unit or if their clinical condition precluded survey completion (e.g. acute illness, delirium, cognitive impairment, or aphasia).

For the present analysis, participants had to be present on the neurology unit at 11 pm and return from any temporary transfers before 6 am Participants with incomplete lights-out and final awakening times were excluded, and only the first night of survey data was included for those who completed multiple surveys during the same hospitalization to maintain independence of observations across participants. Of 256 surveyed patients, 247 met analytic criteria after excluding those who did not meet overnight presence requirements (*n* = 3) and those with missing key sleep timing variables (*n* = 6). This study used data collected from the SIESTA-Neuro quality improvement initiative, a sleep-promoting program in the inpatient neurology setting [5, 13, 14]. Informed consent was obtained from all participants before data collection. As a secondary analysis of existing quality improvement data, it was approved by the Research Subjects Review Board at the University of Rochester as exempt research (Study #6345).

### Inpatient sleep experience measures

For **sleep duration**, participants were asked, “How long did you sleep?” Karolinska Sleep Diary (KSD)–reported lights-out and final awakening times were used to define the start and end of the **patient-defined sleep window** [15].

For **sleep quality**, participants completed three KSD items: (1) refreshing sleep, “Did you feel refreshed after you arose this morning?” (1 = not at all to 5 = completely), (2) sound sleep, “Did you sleep soundly?” (1 = very restless to 5 = very soundly), and (3) sleep continuity, “Did you sleep throughout the time allotted for sleep?” (1 = woke up much too early, 5 = slept through the night) [15].

For **sleep disruption** due to hospital-related factors, participants completed the Potential Hospital Sleep Disruptions and Noises Questionnaire (PHSDNQ) [16, 17]. This instrument includes items that assess the severity of disruption on a 1–5 scale across four classes of PSIs: vital sign checks, neurologic checks, medication administration, and diagnostic testing. A composite score, computed as the average of the four individual items, was used as an overall measure of sleep disruption.

Missing data for inpatient sleep experience measures were minimal (<2% for all variables). Correlations were estimated using pairwise complete observations, and no imputation was performed, given the low degree of missingness.

### EHR-derived sleep measures

PSIs were extracted from overnight activity documented in the EHR using structured data sources that capture clinical events in predefined, queryable fields. PSIs represent distinct, time-stamped events that involve bedside care or direct patient interaction. Events were categorized into six categories: vital signs, neurological checks, medication administrations, bedside diagnostic testing, off-unit transfers, and on-unit transfers. Structured EHR data sources used to query PSIs included nursing flowsheets, medication administration records, orders, and admission data transfer records. Event timestamps were derived from either automated system entries (e.g. medication administration following the scanning of patient bracelets and medication packaging) or from point-of-care staff documentation, in line with standard inpatient workflows. Only documented clinical activities were included to best represent overnight patient care interactions.

Each PSI was modeled as a time interval. For imaging studies and off-unit diagnostic testing, start and end times were determined using documented timestamps within the EHR. For other PSIs without documented end times, the start time was set to the documented timestamp, and the end time was assumed to be 5 min later to approximate the interaction duration. Temporary off-unit transfers were not classified as PSIs because they represent prolonged care intervals and frequently overlap with diagnostic testing. However, off-unit transfers were incorporated into the definition of interruptive episodes and factored into the calculation of uninterrupted sleep opportunity.

Interruptive episodes were defined as any PSI, off-unit transfer, or multiples of these events occurring within 20 min of the preceding interaction, with the 20-min threshold resetting dynamically with each new interaction. PSIs and transfers occurring within this window were grouped and treated as a single interruptive episode, reflecting clustered overnight clinical activity. An interruptive episode could therefore include one or more PSIs and could range in duration from a single buffered interaction to an entire night without uninterrupted intervals.

After applying these rules, we derived two additional EHR measures: (1) longest uninterrupted sleep opportunity (LUSO): the longest continuous interval without an interruptive episode during the sleep window (this is either from the start of the night until first episode, between episodes, or after the final episode until the end of the sleep window); and (2) total uninterrupted sleep opportunity (TUSO): the sum of all uninterrupted intervals between interruptive episodes during the sleep window. This rule-based approach to deriving sleep opportunity measures from EHR data has been described in prior studies [8, 9].

### Sleep window definitions

EHR-derived sleep measures were calculated within two distinct time windows. The first was a fixed window from 11 pm to 6 am, based on the study unit’s typical workflow. On this unit, patient care activities, such as vital signs and neurological checks, typically occur around 11 pm, coinciding with shift change for nursing staff. Morning patient care activities begin at approximately 6 am This fixed period, therefore, reflects the routine overnight interval during which we expect patients on this unit to have their main opportunity for sleep.

The second was a patient-defined window based on the lights-out and final awakening times reported in the KSD. This approach aligned EHR-derived sleep measures with each patient’s sleep opportunity period, which could differ from the fixed, unit-based interval. For each window type, we calculated LUSO, TUSO, and nightly PSI counts (vital signs, neurological checks, medications, bedside testing, off-unit diagnostic testing, and on-unit transfers) using previously published methods [8].

Figure 1 illustrates how window definition influences derived sleep measures for the same patient night. Differences in window boundaries determine which PSIs are included and how uninterrupted periods are defined. In this example, the fixed window yields a slightly longer LUSO and lower PSI count, whereas the patient-defined window yields a greater TUSO.

**Figure 1:**
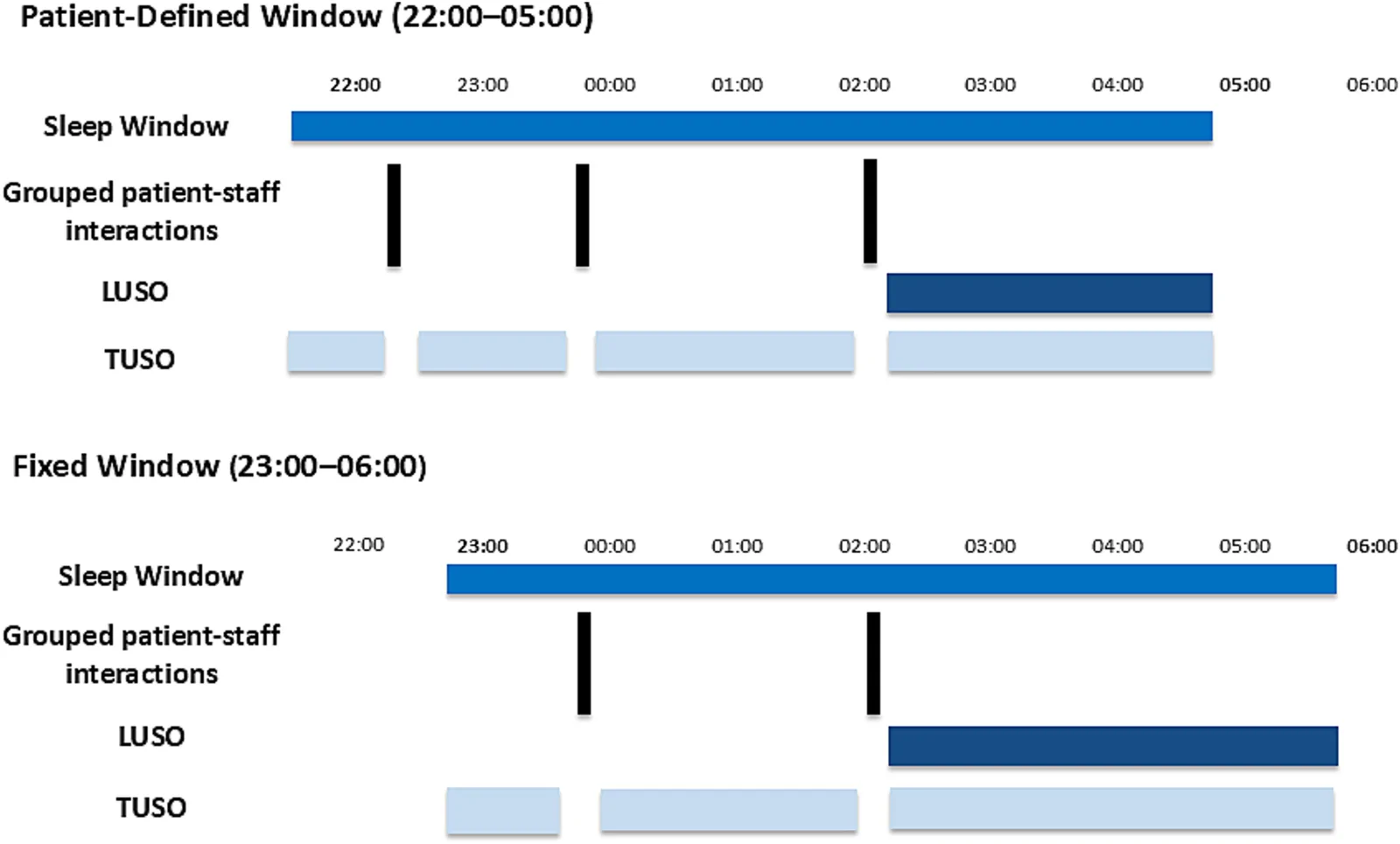
Panels depict the same patient-night under a patient-defined window (22:00–05:00) and a fixed window (23:00–06:00). Patient-staff interactions are shown as vertical bars; fewer interactions fall within the fixed window due to differing boundaries. LUSO represents the single longest uninterrupted period within the window, and TUSO represents the sum of all uninterrupted segments. In this example, the fixed window yields a slightly longer LUSO and lower patient-staff interaction count, whereas the patient-defined window yields a greater TUSO.

### Data analysis

We summarized patient demographics, sleep experience measures, and EHR-derived sleep measures using descriptive statistics. Nonparametric methods were used given that the variables had skewed distributions with normality assumptions not met. Median differences in LUSO, TUSO, and PSI counts between fixed and patient-defined windows were evaluated using the Wilcoxon signed-rank test. We constructed scatterplots to visualize the relationship and evaluate agreement between EHR-derived measures across the two window types. We used Bland–Altman analyses to compare LUSO and TUSO across the two window types.

We used Spearman correlations to examine associations between sleep experience and EHR-derived sleep measures. For both window types, we assessed the ability of: (1) LUSO and TUSO to predict sleep duration; (2) LUSO and TUSO to predict sleep quality measures (refreshing sleep, sound sleep, and sleep continuity); (3) total PSI counts to predict overall sleep disruption from medical interventions; and (4) individual PSI counts (number of vital sign checks, neurological checks, medication administrations, and bedside testing) to predict sleep disruption from those specific interactions.

To assess how well LUSO and TUSO matched the reference standard (sleep duration), we used bias plots. Bias plots are designed for method-comparison studies in which one method serves as the gold standard and shows the systematic difference (bias) between the methods relative to the true value from the reference method [18].

Since PSI counts were not expected to measure the same construct as sleep disruption ratings, we did not use Bland–Altman methods to compare them with sleep experience measures. Instead, we relied on association-based approaches to see if PSI counts predicted perceived sleep disruption. We ran univariable linear regression models with the PHSDNQ medical-intervention composite score as the outcome and PSI counts (vital signs, medications, bedside testing, neurological checks) as predictors. Off-unit and on-unit transfers were not included because the PHSDNQ did not directly ask about these sleep disruptions. These models estimated the average change in the disruption score for each additional nighttime interaction. Analyses were conducted for PSI counts calculated within both window types. Given the focus on construct validity, analyses were not adjusted for additional patient-level factors (e.g. illness severity), as these are closely intertwined with both overnight clinical interactions and patients’ sleep experiences. Formal adjustment for multiple comparisons was not applied, as the emphasis was on the consistency and magnitude of associations.

All regression coefficients (β) are presented with their corresponding 95% confidence intervals (CIs). Statistical significance was defined a priori as *p* < .05. Analyses were performed using SAS 9.4 and R version 4.2.3.

## Results

### Cohort characteristics

The study included 247 inpatients hospitalized on the neurology unit. The median (IQR) age was 55 (38, 70) years; 52% were female, and 82% were white. Approximately half of the inpatient nights (51%) were in shared rooms. The most common primary diagnoses were ischemic stroke (63 [25.5%]), epilepsy (54 [21.9%]), and hemorrhagic stroke (19 [7.7%]). Median length of stay was 5.9 days (IQR, 4.0–11.0).

For KSD measures, the median (IQR) for sleep duration was 6.0 h (4.9 h, 8.0 h), lights-out time was 22:00 (19:45, 22:30), final awakening time was 7:00 (6:00, 8:00), and total sleep opportunity, or the patient-defined sleep window, was 8.5 h (7.0 h, 10.0 h).

For sleep quality measures, the median (IQR) scores were 3 (1, 4) for refreshing sleep, 3 (2, 5) for sound sleep, and 3 (1, 5) for sleep continuity, indicating that most participants rated their sleep quality as average. Sleep disruption from medical interventions was generally not disruptive to somewhat disruptive (PHSDNQ composite median 1.5; IQR 1.0, 2.3).

### Descriptive statistics of EHR-derived sleep measures and comparison of windows

Patient-defined windows were generally longer than the 7-h window assumed by the fixed window approach, with earlier lights-out and later final awakening times.

Median PSI counts were lower within the fixed window (2 [0, 4] vs. 3 [1, 6]; *p* < .001). Histograms demonstrating the distribution of PSIs per patient-night for both fixed and patient-defined windows are shown in Figure S1. Vital sign checks, medication administrations, and neurological checks generally occurred more frequently within patient-defined windows.

Median (IQR) LUSO was slightly longer when calculated within the fixed window compared to the patient-defined window (5.2 h [3.7, 7.0] vs. 5.0 h [3.7, 7.1], *p* = .006). Although patient-defined LUSO exceeded fixed LUSO in many pairwise comparisons (Figure 2), differences were typically larger when fixed LUSO was greater (points farther from the line of identity), resulting in a modestly higher median. In these cases with larger differences, the patient-defined window ended earlier, allowing the fixed window to capture a longer early-morning LUSO (Figure 1).

**Figure 2:**
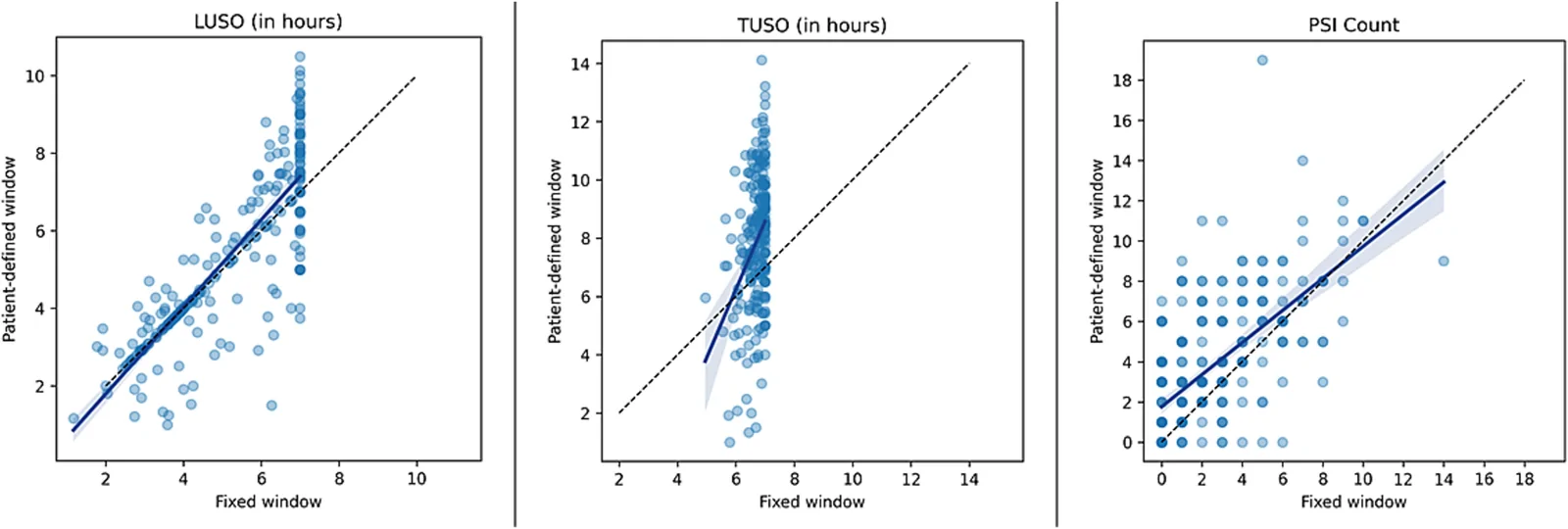
Scatterplots of patient-defined vs. fixed windows for EHR-derived sleep measures. Scatterplots comparing LUSO, TUSO, and patient-staff interaction (PSI) counts calculated within patient-defined vs. fixed windows across 247 patients. The dashed line represents the line of identity. The solid line represents the regression line, and the shaded band represents the 95% confidence interval. Darker shading of the data points indicates greater overlap, reflecting multiple observations at similar values.

Median TUSO was substantially shorter within the fixed window (6.8 h [6.5, 7.0] vs. 8.0 h [6.6, 9.4], *p* < .001).

Scatterplots comparing fixed and patient-defined windows (Figure 2) demonstrate a strong correlation for LUSO, with values clustering tightly along the line of identity and a visible ceiling effect at 420 min, corresponding to the fixed window cap. TUSO showed greater dispersion, with patient-defined TUSO spanning a wider range. Variability was most pronounced at shorter sleep opportunities, as indicated by a wider confidence band in the scatterplot. In contrast, the LUSO scatterplot demonstrated a consistently narrow confidence band across durations. PSI counts were positively correlated across windows, with greater variability at higher counts.

Bland–Altman analyses comparing the fixed and patient-defined window approaches (Figure 3) demonstrated a small mean bias for LUSO (−10 min) but a significant proportional bias (β₁ = -0.32 h per h, *p* < .001), indicating greater underestimation by the fixed window approach at higher LUSO values. A larger mean bias (−71 min) was observed in the two approaches to measuring TUSO, with wider limits of agreement and higher proportional bias (β₁ = -1.74 h per h, *p* < .001).

**Figure 3:**
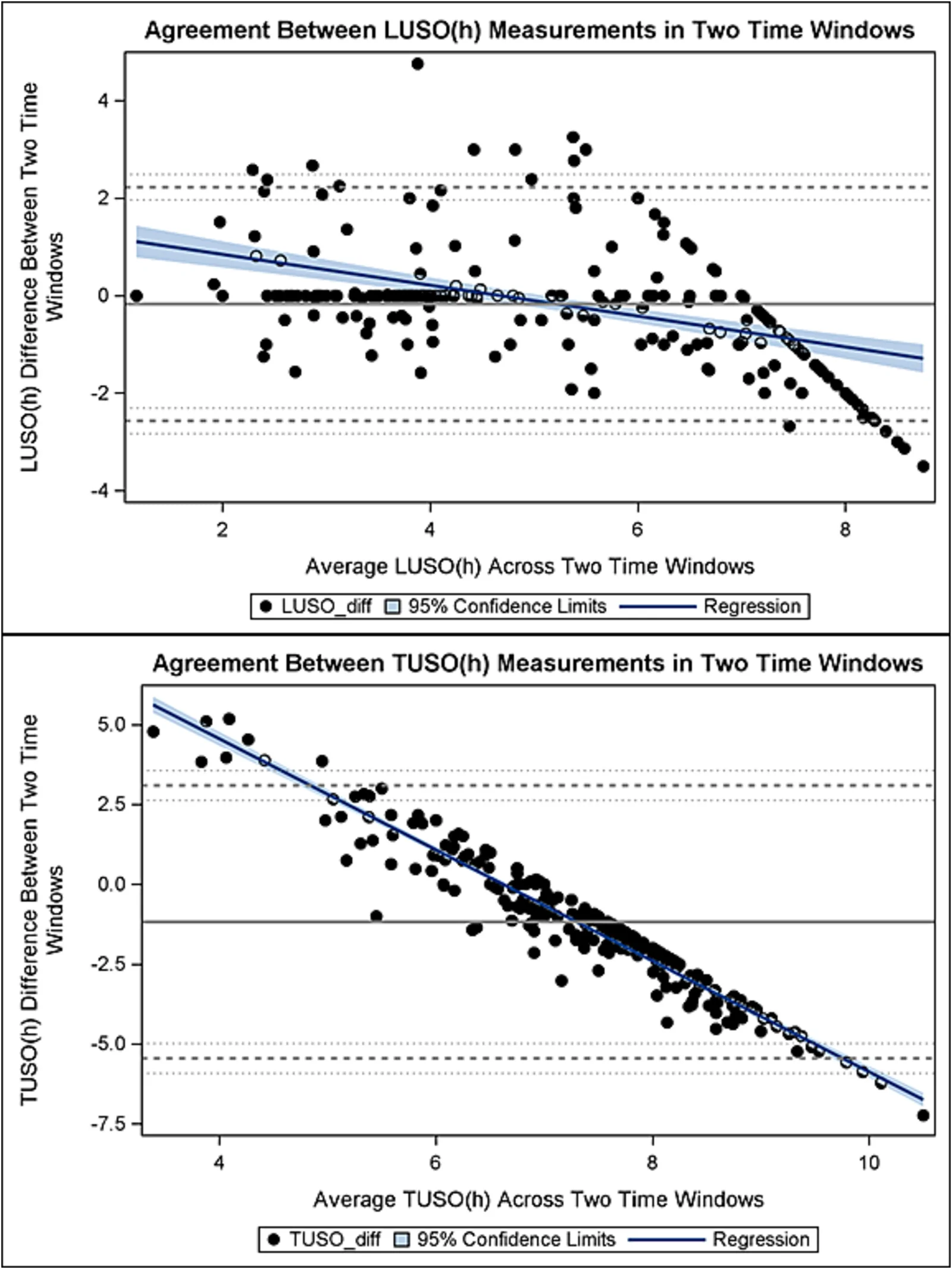
Bland–Altman plots comparing fixed and patient-defined windows for EHR-derived sleep opportunity measures. Bland–Altman plots for the LUSO and the TUSO. Differences were calculated as the fixed window minus the patient-defined window. The solid line represents the mean difference (bias), and the dashed lines represent the 95% limits of agreement.

### Validation of EHR-derived sleep measures

We evaluated how well the EHR-derived measures captured patients’ sleep experiences across both window types (Table 1).

**Table 1:** Correlations of electronic health record-derived sleep variables with patient sleep experience measures

**Patient sleep experience measure**	**EHR-derived sleep variable**	**Fixed window**		**Patient-defined window**	
		***r***	***P* value**	***r***	***P* value**
**Sleep duration**	LUSO	0.20	.002	0.43	<.001
	TUSO	0.27	<.001	0.63	<.001
**Sleep continuity**	LUSO	0.02	.74	0.15	.02
	TUSO	0.12	.06	0.23	<.001
	Total PSI Count	−0.07	.26	0.04	.50
**Sound sleep**	LUSO	0.02	.75	0.09	.16
	TUSO	0.05	.43	0.19	.003
**Refreshing sleep**	LUSO	−0.08	.23	0.01	.89
	TUSO	0.06	.39	0.15	.016
**Sleep disruption from medical interventions**	Total PSI count	0.35	<.001	0.29	<.001

Values represent Spearman correlation coefficients (*r*) and corresponding *p* values.

Abbreviations: EHR: electronic health record, LUSO: longest uninterrupted sleep opportunity, TUSO: total uninterrupted sleep opportunity, PSI: patient–staff interaction.

**Sleep duration** was significantly associated with both LUSO and TUSO, supporting the construct validity of these EHR-based sleep measures. TUSO showed the highest correlations with sleep duration. Furthermore, correlations were twice as strong when TUSO was calculated within the patient-defined window (*r* = 0.63; both *p* < .001) than with the fixed window approach (*r* = 0.27; *p* < .001). **Sleep continuity** was also significantly associated with both LUSO and TUSO, with the strongest correlation within the patient-defined window for TUSO (*r* = 0.23; *p* < .001), further supporting its construct validity. Notably, **sound sleep** and **refreshing sleep** were associated with TUSO only in the patient-defined window (*r* = 0.19, *p* = .003; *r* = 0.15, *p* = .016, respectively).

**Sleep disruption from medical interventions**, measured by the composite PHSDNQ, was significantly associated with total PSI count, indicating that greater overnight activity was associated with higher disruption ratings. The association was slightly stronger in the fixed window (*r* = 0.35 vs. 0.29; both *p* < .001).

When **sleep disruption** was examined by medical intervention category, each domain score was significantly associated with the frequency of its corresponding PSI. The window choice had little impact on disruption of bedside testing (*r* = 0.33 vs. 0.34, both *p* < .001) and medication administration (both *r* = 0.22, *p* < .001). In contrast, associations with vital sign disruption were stronger when *vital sign counts* were calculated within the fixed window, while neurological check disruption was significant only when *neurological check counts* were calculated within the patient-defined window (Table 2).

**Table 2:** Correlations of sleep disruption and corresponding electronic health record–derived patient–staff interaction

**Sleep disruption**	**EHR-derived PSI**	**Fixed window**		**Patient-defined window**	
		***r***	***P* value**	***r***	***P* value**
**Vital sign disruption**	Vital sign count	0.27	<.001	0.20	.001
**Neuro disruption**	Neuro check count	0.09	.14	0.16	.014
**Medication**					
**Disruption**	Medication count	0.22	<.001	0.22	<.001
**Testing disruption**	Bedside testing count	0.33	<.001	0.34	<.001

For each correlation, we used the PHSDNQ sleep disruption domain and the corresponding EHR-documented patient–staff interaction count, calculated within the fixed and patient-defined windows. Values represent Spearman correlation coefficients (*r*) and corresponding *p* values.

Abbreviations: EHR: electronic health record, PSI: patient–staff interaction, PHSDNQ: Potential Hospital Sleep Disruptions and Noises Questionnaire.

### Bias between EHR-derived and sleep experience measures

Bias plot analyses (Figure 4) comparing EHR-derived sleep opportunity measures with sleep duration (treated as the reference standard) demonstrated significant proportional bias under fixed windows for both LUSO (β₁ = −0.87 h per h, *p* < .0001) and TUSO (β₁ = −0.96 h per h, *p* < .0001). Patient-defined sleep windows reduced proportional bias for both LUSO (β₁ = −0.62 h per h, *p* < .0001) and TUSO (β₁ = −0.42 h per h, *p* < .0001). Although patient-defined TUSO had a larger positive mean bias (95 min), it showed the smallest proportional bias and the narrowest limits of agreement.

**Figure 4:**
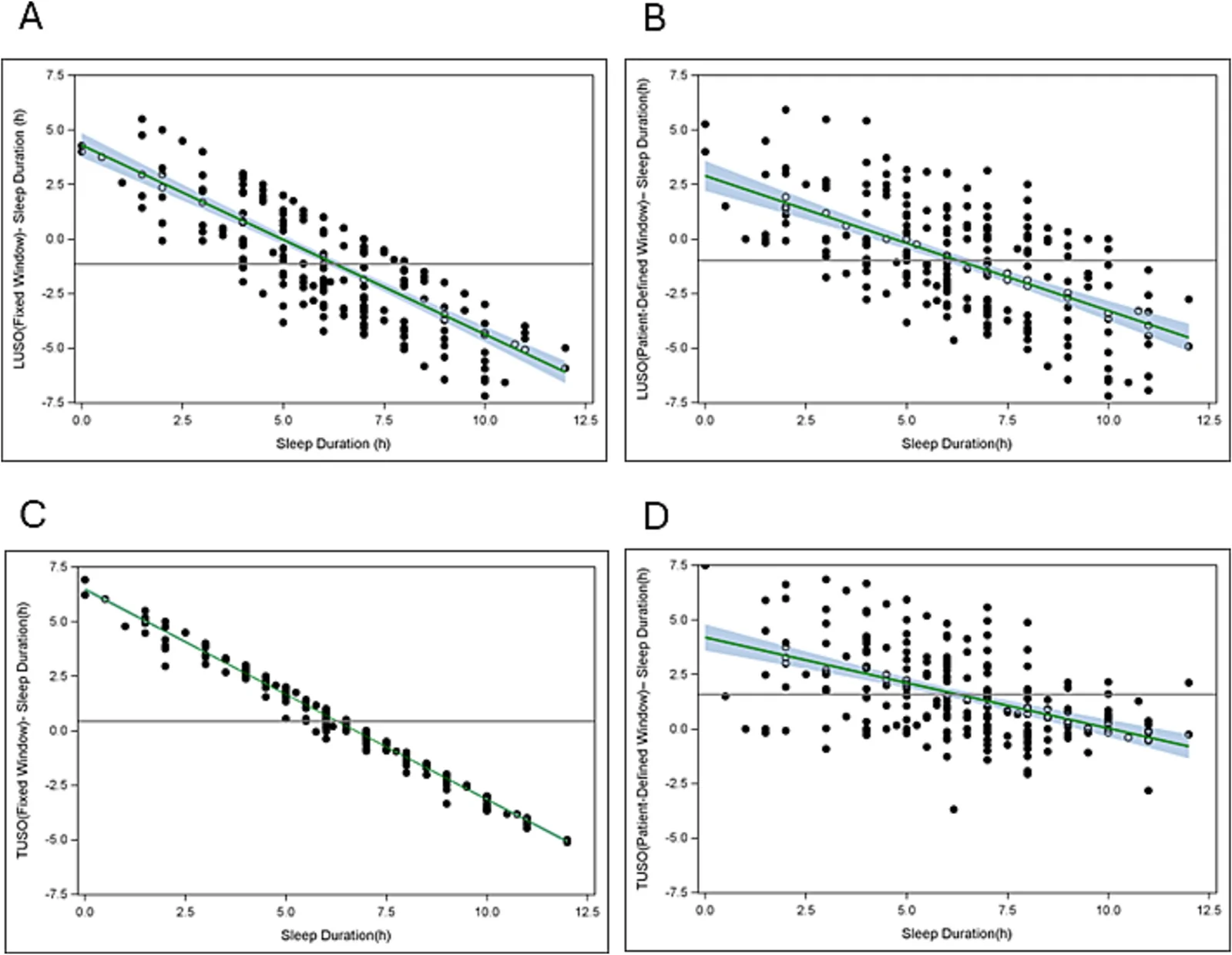
Bias plots comparing EHR-derived sleep opportunity measures with self-reported sleep duration (reference standard). (A) Longest uninterrupted sleep opportunity calculated within the fixed window; (B) longest uninterrupted sleep opportunity calculated within the patient-defined window; (C) total uninterrupted sleep opportunity calculated within the fixed window; and (D) total uninterrupted sleep opportunity calculated within the patient-defined window. Bias was calculated as the difference between EHR-derived sleep opportunity and self-reported sleep duration. Solid lines represent the linear regression of bias on sleep duration. Slopes closer to zero indicate reduced proportional bias.

Mean absolute error (MAE) and root mean square error analyses comparing EHR-derived measures with self-reported sleep duration showed lower error for TUSO than LUSO across both window definitions, with patient-defined TUSO demonstrating the lowest MAE (Table S1).

### Regression analysis of EHR-derived sleep measures

#### PSI counts (vital sign checks, medication administrations, bedside testing, and neurological checks) as predictors of sleep disruption

We evaluated the mean changes in the PHSDNQ Medical Intervention Composite Score per additional EHR-documented PSI (Table 3). Bedside testing consistently emerged as the strongest predictor across both windows, with each additional bedside test associated with an approximately 0.6-point increase on the 5-point disruption scale.

**Table 3:** Electronic health record—derived patient–staff interactions as predictors of sleep disruption (PHSDNQ)

**Patient–staff interaction type**	**Fixed window**		**Patient-defined window**	
	**Change in sleep disruption (95% CI)**	***P*-value**	**Change in sleep disruption (95% CI)**	***P*-value**
**Vital sign checks**	0.29 (0.15 to 0.44)	.0001	0.14 (0.02 to 0.26)	.025
**Neuro checks**	0.22 (0.06 to 0.37)	.005	0.26 (0.12 to 0.39)	.0003
**Medication administrations**	0.19 (0.10 to 0.28)	<.0001	0.13 (0.06 to 0.19)	.0002
**Bedside testing**	0.64 (0.39 to 0.88)	<.0001	0.59 (0.35 to 0.83)	<.0001

Values represent model-estimated mean changes in PHSDNQ (Medical Intervention Composite) scores per additional EHR-documented patient–staff interaction within each sleep window.

Abbreviations: PHSDNQ = Potential Hospital Sleep Disruptions and Noises Questionnaire, CI = confidence interval.

### Exploratory subgroup analyses

Exploratory subgroup analyses showed consistent associations between EHR-derived sleep opportunity metrics and sleep duration across race, gender, and age groups. When stratified by race, correlations between sleep duration and both LUSO and TUSO were similar across groups. Within the fixed window, sleep duration correlated with LUSO (white: *r* = 0.19; non-white: *r* = 0.23) and TUSO (white: *r* = 0.26; non-white: *r* = 0.35), and in the patient-defined window with LUSO (white: *r* = 0.41; non-white: *r* = 0.49) and TUSO (white: *r* = 0.60; non-white: *r* = 0.72), with no statistically significant difference between groups (all *p-*values >.60).

Findings were similarly consistent across gender and age groups. Within the patient-defined window, sleep duration correlated with TUSO in both males (*r* = 0.60) and females (*r* = 0.68) and with LUSO in males (*r* = 0.48) and females (*r* = 0.37); correlations within the fixed window were also comparable across genders (*r* = 0.11 – 0.27). Likewise, correlations between sleep duration and patient-defined TUSO (≥55 years: *r* = 0.62, <55 years: *r* = 0.66) and LUSO (≥55 years: *r* = 0.46, <55 years: *r* = 0.39) were similar across age groups, with comparable correlations observed within the fixed window (*r* = 0.15–0.29).

Associations between EHR-derived PSIs and perceived sleep disruption from medical interventions were also comparable across subgroups. Using the fixed window, correlations between total PSI count and PHSDNQ composite score were similar by race (white: *r* = 0.34; non-white: *r* = 0.37), gender (male: *r* = 0.35; female: *r* = 0.33), and age (≥55 years: *r* = 0.28; <55: *r* = 0.39) with no statistically significant differences between groups (all *p*-values >.65). Results were similar when using the patient-defined sleep window (race: white *r* = 0.29; non-white *r* = 0.36, gender: male *r* = 0.29; female *r* = 0.28; age: ≥55 years *r* = 0.18; <55 years *r* = 0.39, all *p*-values >.40).

## Discussion

Sleep in hospitalized patients is increasingly recognized as clinically important for optimizing clinical recovery, yet it is rarely measured. This study addresses that gap by using scalable approaches based on routinely collected clinical data to evaluate whether EHR-derived measures of sleep opportunity and interruption meaningfully reflect patients’ reported sleep experiences. Greater EHR-derived sleep opportunities, particularly TUSO, and lower PSIs were associated with better patient-reported sleep, supporting the construct validity of these measures. Among individual PSIs, bedside testing was associated with the highest perceived sleep disruption.

When we compared a fixed window based on unit workflow (11 pm to 6 am) with patient-defined sleep windows using sleep diaries, we found that uninterrupted sleep opportunity measures, especially TUSO, were more strongly associated with sleep duration and different aspects of sleep quality, such as how continuous, refreshing, and sound their sleep was. In contrast, sleep opportunity measures within the fixed window were not correlated with any KSD sleep quality ratings. Bias analyses comparing LUSO and TUSO with sleep duration showed proportional bias with fixed windows, but this bias was reduced with patient-defined windows. Together, these findings suggest that incorporating patient-defined sleep timing improves the accuracy of EHR-derived opportunity measures in reflecting sleep duration and multiple dimensions of sleep quality.

In contrast, PSIs within fixed time windows demonstrated stronger or equivalent associations with perceived sleep disruption as measured by the PHSDNQ. The total PSI count was moderately correlated with overall perceived disruption, with this correlation slightly higher in the fixed window. Individual PSI counts, including vital signs, medication administrations, and bedside testing, also showed stronger or equivalent associations with perceived disruption in fixed windows than in patient-defined windows.

One possible explanation lies in sleep onset, which is a gradual, continuous process rather than a discrete event. During this transition, individuals experience fluctuating states between wakefulness and sleep, during which they remain sensitive to external stimuli [19]. PSIs in this transition stage may lead patients to perceive that they have not yet fallen asleep, delaying the perceived onset of sleep and excluding initial interactions that still disrupt sleep. In contrast, fixed windows align with overnight clinical workflows, consistently capturing disruption events independent of patients’ perceptions of sleep onset or offset.

These fixed window findings align with and extend our prior work on cardiac surgery inpatients, in which TUSO demonstrated stronger associations with sleep quality, as assessed by Richards-Campbell Sleep Questionnaire (RCSQ) mean scores, than either LUSO or interruptive episode counts. Lower TUSO values calculated within a fixed overnight window were associated with worse sleep quality, as measured by the RCSQ. In that study, the absence of sleep diaries necessitated a fixed window approach [9]. The RCSQ evaluates domains such as awakenings from sleep and the ability to return to sleep, which may align well with fixed time windows. By contrast, the KSD emphasizes these domains less and may be less compatible with EHR-derived measures. This hypothesis warrants further evaluation in other inpatient populations.

### Interpretation and implications

Whereas EHR-derived sleep opportunity measures calculated within patient-defined windows were more strongly associated with sleep duration, demonstrating higher correlations, reducing proportional bias, and narrowing limits of agreement in bias analyses, various mean differences relative to self-reported sleep duration persisted. Across windowing strategies, TUSO was 16 to 90 min longer than self-reported sleep duration. In contrast, LUSO was 60 to 76 min shorter than sleep duration, indicating that these measures may over- and underestimate perceived sleep duration.

Differences between objective and self-reported sleep duration are not unique to EHR-based approaches. Prior studies comparing self-reported sleep duration with actigraphy and polysomnography similarly demonstrate differences. For example, one population-based study found that individuals with shorter actigraphy-measured sleep durations estimate longer sleep durations than those with longer actigraphy-measured sleep durations (a difference of 1.2 h vs. 0.4 h) [20]. Other population-based studies have found that individuals estimated their habitual sleep duration to be 20–70 min longer than both PSG- and actigraphy-assessed sleep duration [21, 22].

Notably, subjective sleep is recognized as a distinct and clinically meaningful dimension of sleep health [23]. Epidemiologic data demonstrate that self-reported and polysomnography-measured sleep duration show distinct associations with mortality [24], and that the subjective experience of sleep restfulness independently modifies this relationship [25], suggesting patient-reported sleep captures prognostic information beyond physiologic measurement alone. That our EHR-derived measures demonstrate construct validity against this dimension is therefore clinically relevant, as it suggests these measures may capture aspects of the inpatient sleep experience that matter to patients and predict outcomes independent of physiologic sleep architecture.

To our knowledge, no published studies have formally evaluated the agreement or correlation between sleep diary and actigraphy measurements in general inpatient populations. Given that the hospital environment may uniquely influence both subjective sleep perception and the accuracy of objective sleep measurements, a formal validation study examining agreement among sleep diary, actigraphy, and EHR measures in general medical and surgical inpatient populations represents an important opportunity for future research.

### Translation to practice

A practical consideration is how hospital staff may collect patient-defined and fixed windows in real-world inpatient settings. Hospital staff or patients themselves could provide patient-defined sleep windows via brief daily logs or single-item questions embedded in nursing assessments or patient portals (e.g. “What time did you try to fall asleep?” and “What time did you wake up for the last time?”). Information technology teams could integrate such functionality into existing platforms that already deliver questionnaires to inpatients. Knowing a patient’s intended bedtime would allow staff to complete routine tasks in advance, thereby reducing avoidable nighttime disruptions. Nurses and physicians could adjust medication timing, bedside testing, or assessments within a patient’s declared sleep window while still ensuring safety. Morning reports of wake times could then improve the calculation of EHR-derived estimates of sleep opportunity and fragmentation in real time.

Fixed windows remain highly pragmatic, particularly for quality improvement initiatives or hospital-wide surveillance that cannot rely on patient input. Notably, prior studies using EHR-derived sleep measures have used fixed nighttime windows but have not described how these intervals were chosen, leaving unclear whether they reflect patient experience or workflow realities. In our study unit, we selected 11 pm to 6 am as the fixed window, based on discussions with staff that revealed the shift change occurred at 11 pm, when nurses typically awakened patients for assessments, and that morning rounds began at 6 am. However, the observed mismatch between fixed and patient-defined windows suggests that such standardized intervals may not fully align with patients’ actual sleep opportunities. While similar engagement with frontline staff could inform fixed window selection in other settings, using aggregated patient-defined data could be used to refine these standardized windows, creating an iterative process that combines patient-centered and workflow perspectives.

### Limitations

This study has several limitations. First, we obtained patient-defined sleep windows retrospectively in the morning, which introduces potential recall bias and imprecision in the reported lights-out and final awakening times. Second, the sleep questionnaires used have not been formally validated in general inpatient populations, which may affect interpretability in this setting. Third, we did not use polysomnography, which precludes validation of EHR-derived measures against objective sleep measures such as total sleep time and wake after sleep onset. Our findings, therefore, represent construct validity against the patient-reported sleep experience rather than criterion validity against physiologic sleep. However, we aimed to validate scalable, patient-centered EHR-derived measures that could be applied across hospital settings, a strength of this work. Fourth, we conducted this analysis in a single inpatient neurology unit, and workflows that influence the fixed window may differ across hospitals and units, limiting generalizability. Fifth, because analyses were limited to a single night per participant, findings may not generalize to sleep patterns across the entire hospitalization. Finally, unmeasured factors such as illness severity, anxiety, stress, or environmental disturbances may have influenced sleep experience and EHR-derived measures.

## Conclusion

EHR-derived measures of sleep opportunity and interruption offer a scalable approach to characterizing the inpatient sleep experience using routinely collected clinical data. Our findings indicate that patient-defined windows more accurately capture the duration and quality of sleep opportunities, whereas fixed windows are equally effective at assessing sleep disruption associated with overnight clinical workflows. These complementary approaches provide valuable insights into inpatient sleep and can inform future strategies to minimize unnecessary nighttime disturbances and enhance sleep quality. Integrating EHR-derived metrics with objective sleep monitoring and patient-reported outcomes will be essential to refining these measures and guiding sleep-friendly hospital practices. Future work should also determine how these EHR-derived sleep measures relate to patient recovery, safety, and satisfaction.

## Supplementary Material

Supplementary_File_zsag164

## Data Availability

The code for data processing and analysis is publicly available at: https://github.com/lisapink-urmc/SLEEP-Defining-the-Hospital-Night.

## Notes

Address where work was conducted: Strong Memorial Hospital, University of Rochester Medical Center, 601 Elmwood Avenue, Rochester, NY 14642, USA

## References

[1] Mansour W, Knauert M. Adding insult to injury: sleep deficiency in hospitalized patients. *Clin Chest Med*. 2022;43(2):287–303. 10.1016/j.ccm.2022.02.009 PMC9177053 35659026

[2] Stewart NH, Arora VM. Sleep in hospitalized older adults. *Sleep Med Clin*. 2018;13(1):127–135. 10.1016/j.jsmc.2017.09.012 PMC5880551 29412979

[3] Redeker NS, Ruggiero JS, Hedges C. Sleep is related to physical function and emotional well-being after cardiac surgery. *Nurs Res*. 2004;53(3):154–162. 10.1097/00006199-200405000-00002 15167503

[4] Wesselius HM, van den Ende ES, Alsma J, et al. Quality and quantity of sleep and factors associated with sleep disturbance in hospitalized patients. *JAMA Intern Med*. 2018;178(9):1201–1208. 10.1001/jamainternmed.2018.2669 PMC6142965 30014139

[5] Kadura S, Poulakis A, Roberts DE, et al. Sleeping with one cerebrum open: patient and staff perceptions of sleep quality and quantity on an inpatient neurology unit. *J Clin Sleep Med*. 2023;20(4):619–629. 10.5664/jcsm.10958 PMC10985310 38063214

[6] Ruben MD, Francey LJ, Guo Y, et al. A large-scale study reveals 24-h operational rhythms in hospital treatment. *Proc Natl Acad Sci U S A*. 2019;116(42):20953–20958. 10.1073/pnas.1909557116 PMC6800314 31575744

[7] Schwab KE, Ronish B, Needham DM, To AQ, Martin JL, Kamdar BB. Actigraphy to evaluate sleep in the intensive care unit. A systematic review. *Ann Am Thorac Soc*. 2018;15(9):1075–1082. 10.1513/AnnalsATS.201801-004OC PMC6322043 29944386

[8] Kadura S, Pink L, Mazzotti DR, Fear K. Evaluating the impact of patient-staff interactions on sleep opportunities using the electronic health record. *J Hosp Med*. 2024;20(6):581–587. 10.1002/jhm.13568 39632392

[9] Oldham MA, Pink L, Kadura S. Electronic health record-derived measures of sleep after surgical aortic valve replacement. *Am J Crit Care*. 2026;35(3):205–213. 10.4037/ajcc2026195 42062214

[10] Catley CD, Paynter K, Jackson K, et al. Redesigning the hospital environment to improve restfulness. *JAMA Netw Open*. 2024;7(12):e2447790. 10.1001/jamanetworkopen.2024.47790 PMC11618460 39630451

[11] Najafi N, Robinson A, Pletcher MJ, Patel S. Effectiveness of an analytics-based intervention for reducing sleep interruption in hospitalized patients: a randomized clinical trial. *JAMA Intern Med*. 2022;182(2):172–177. 10.1001/jamainternmed.2021.7387 PMC8715385 34962506

[12] Haimovich AD, Bertisch SM, Jegadeesan V, Stevens JP, Schonberg MA, Berry SD. Sleep interruptions among older adults admitted to the hospital. *JAMA Netw Open*. 2025;8(3):e251131. 10.1001/jamanetworkopen.2025.1131 PMC11923702 40105845

[13] Kadura S, Eisner L, Lopa SH, et al. Nudging towards sleep-friendly health care: a multifaceted approach on reducing unnecessary overnight interventions. *Appl Clin Inform*. 2024;15(5):1025–1039. 10.1055/a-2404-2344 PMC11602248 39191425

[14] Arora VM, Machado N, Anderson SL, et al. Effectiveness of SIESTA on objective and subjective metrics of Nighttime hospital sleep disruptors. *J Hosp Med*. 2019;14(1):38–41. 10.12788/jhm.3091 30667409

[15] Akerstedt T, Hume K, Minors D, Waterhouse J. The meaning of good sleep: a longitudinal study of polysomnography and subjective sleep quality. *J Sleep Res*. 1994;3(3):152–158. 10.1111/j.1365-2869.1994.tb00122.x 10607120

[16] Grossman MN, Anderson SL, Worku A, et al. Awakenings? Patient and hospital staff perceptions of nighttime disruptions and their effect on patient sleep. *J Clin Sleep Med*. 2017;13(2):301–306. 10.5664/jcsm.6468 PMC5263086 27923432

[17] Erondu AI, Orlov NM, Peirce LB, et al. Characterizing pediatric inpatient sleep duration and disruptions. *Sleep Med*. 2019;57:87–91. 10.1016/j.sleep.2019.01.030 PMC6760863 30921685

[18] Taffe P . Effective plots to assess bias and precision in method comparison studies. *Stat Methods Med Res*. 2018;27(6):1650–1660. 10.1177/0962280216666667 27705883

[19] Lacaux C, Strauss M, Bekinschtein TA, Oudiette D. Embracing sleep-onset complexity. *Trends Neurosci*. 2024;47(4):273–288. 10.1016/j.tins.2024.02.002 38519370

[20] Lauderdale DS, Knutson KL, Yan LL, Liu K, Rathouz PJ. Self-reported and measured sleep duration: how similar are they? *Epidemiology.* 2008;19(6):838–845. 10.1097/EDE.0b013e318187a7b0 PMC2785092 18854708

[21] Matthews KA, Patel SR, Pantesco EJ, et al. Similarities and differences in estimates of sleep duration by polysomnography, actigraphy, diary, and self-reported habitual sleep in a community sample. *Sleep Health*. 2018;4(1):96–103. 10.1016/j.sleh.2017.10.011 PMC5771411 29332687

[22] Jackson CL, Patel SR, Jackson WB 2nd, Lutsey PL, Redline S. Agreement between self-reported and objectively measured sleep duration among white, black, Hispanic, and Chinese adults in the United States: Multi-Ethnic Study of Atherosclerosis. *Sleep*. 2018;41(6):zsy057. 10.1093/sleep/zsy057 PMC5995218 29701831

[23] Buysse DJ . Sleep health: can we define it? Does it matter? *Sleep.* 2014;37(1):9–17. 10.5665/sleep.3298 PMC3902880 24470692

[24] Zhao B, Meng Y, Jin X, et al. Association of objective and self-reported sleep duration with all-cause and cardiovascular disease mortality: a community-based study. *J Am Heart Assoc*. 2023;12(6):e027832. 10.1161/JAHA.122.027832 PMC10111539 36892074

[25] Yoshiike T, Utsumi T, Matsui K, et al. Mortality associated with nonrestorative short sleep or nonrestorative long time-in-bed in middle-aged and older adults. *Sci Rep*. 2022;12(1):189. 10.1038/s41598-021-03997-z PMC8741976 34997027

