# Supplementary material for: Defining the hospital night: deriving and validating electronic health record-based measures of the inpatient sleep experience
Source: Sleep. 2026 Jun 15;49(10):zsag164. doi: 10.1093/sleep/zsag164 (PMC13658259; doi:10.1093/sleep/zsag164)
Supplement: Supplementary file 1 [file supplementary_file_zsag164.docx]

**Title**: Defining the Hospital Night: Deriving and Validating Electronic Health Record-Based Measures of the Inpatient Sleep Experience

**Authors**: Sullafa Kadura^1,2,7^, Samia H. Lopa^3^, Mark Oldham ^4^, Lisa Pink^2^, Diego R. Mazzotti ^5,6^, Sanjay R. Patel^7^

**Affiliations:**

^1^Department of Medicine, University of Rochester Medical Center, Rochester, NY, USA

^2^University of Rochester Health Lab, University of Rochester Medical Center, Rochester, NY, USA

^3^ Department of Biostatistics and Computational Biology, University of Rochester, Rochester, NY, USA

^4^ Department of Psychiatry, University of Rochester Medical Center, Rochester, NY, USA

^5^Division of Medical Informatics, Department of Internal Medicine, University of Kansas Medical Center, KS, USA

^6^Division of Pulmonary Critical Care and Sleep Medicine, Department of Internal Medicine, University of Kansas Medical Center, KS, USA

^7^Center for Sleep and Cardiovascular Outcomes Research, Division of Pulmonary, Allergy, Critical Care, and Sleep Medicine, University of Pittsburgh, Pittsburgh, PA, USA

**Address Where Work Was Conducted:**

Strong Memorial Hospital
University of Rochester Medical Center
601 Elmwood Avenue
Rochester, NY 14642, USA

**Corresponding author:**

Sullafa Kadura, MD, MBA

UPMC Montefiore, NW628

3459 Fifth Avenue

Pittsburgh, PA 15213

kaduras@upmc.edu

**Table S1. Mean Absolute Error (MAE) and Root Mean Square Error (RMSE) Comparing EHR-Derived Sleep Opportunity Measures with Self-Reported Sleep Duration**

| **Sleep Window**  **Definition** | **Comparison** | **MAE (hours)** | **RMSE (hours)** |
| --- | --- | --- | --- |
| Fixed window | Self-reported sleep duration  vs LUSO | 2.34 | 2.90 |
| Fixed window | Self-reported sleep duration  vs TUSO | 1.92 | 2.42 |
| Patient-defined window | Self-reported sleep duration  vs LUSO | 2.09 | 2.68 |
| Patient-defined window | Self-reported sleep duration  vs TUSO | 1.85 | 2.56 |

**Supplementary Figure Captions:**

**Supplementary Figure S1. Distribution of overnight patient–staff interaction (PSI) counts per patient-night across PSI categories under fixed and patient-defined sleep windows.** Histograms show the distribution of overnight PSI counts for vital signs, medication administrations, bedside testing, neurological checks, off-unit testing, and transfers. PSIs were identified from EHR-documented clinical interactions
